# Supplementary material for: Morphological, ultrastructural and molecular variations in susceptible and resistant genotypes of chickpea infected with Botrytis grey mould
Source: PeerJ. 2023 Mar 28;11:e15134. doi: 10.7717/peerj.15134 (PMC10064989; doi:10.7717/peerj.15134)
Supplement: Supplemental Information 1 [file peerj-11-15134-s001.docx]

**Supplemental Table S1**: List of primers used to screen botrytis responsive genes in PBG5 and *C.pinatifidum*

| **S. No.** | **Primer name** | **Sequence** |
| --- | --- | --- |
| 1 | **BIG 1F**  **BIG 1R** | **5´**ATGTCCTTTATTGGAGAAGCATC **3´**  **3´**CTAAAAGTTATGAATGAACTCACT **5´** |
| 2 | **LrWRKY12 F**  **LrWRKY12 R** | **5´**AGCTTATGTCCGCACTGACC **3´**  **3´**CGCATAGGAACATCTATAGTAAGC **5´** |
| 3 | **LrWRKY4 F**  **LrWRKY4 R** | **5´**CCTCAACAATCAACCATCTTC  **3´**  **3´**CACAGTCTCCTCCTTCAAC **5´** |
| 4 | **W4 F1**  **W4 R1** | **5´**ATGGAGAGCTTCCCCCTACTCCTCAA  **3´**  **3´**TCAAAAGCTCGAATAGACTTGCATTTGG **5´** |
| 5 | **W12 F1**  **W12 R1** | **5´**ATGGATTACTCATATTCTTCATGGATG **3´**  **3´**TCAATCAGAGAATGGCCCATCAGGC **5´** |

**Table S2:** Stomatal index of PBG5 (susceptible) and *C. pinnatifidum* (resistant) genotypes of chickpea

| **S.No.** | **Name of Source** | **No. of Stomata (S)** | **No. of epidermal cells (E)** | **Stomatal index (SI)(%)** |
| --- | --- | --- | --- | --- |
| 1. | PBG5 | 6.9 | 25.5 | 7.17 |
| 2. | *C. pinnatifidum* | 2.3 | 21.0 | 2.41 |
